# Treatment of Club-foot by Means of the Thomas Wrench, with Report of Cases

**Published:** 1889-02

**Authors:** V. P. Gibney

**Affiliations:** New York


### TREATMENT OF CLUB-FOOT BY MEANS OF THE THOMAS WRENCH, WITH REPORT OF CASES.*

By V. P. GIBNEY, M. D., New York.

Mr. President and Gentlemen of the Association: I desire simply to report some cases, with presentation of the apparatus employed, which is known as the Thomas wrench, for forcibly correcting club-foot at one sitting, or, if that is not necessary, take other sittings. The apparatus consists simply of a modified monkey-wrench on a large scale, with arms jutting out from the side, which arms are controlled by a screw, and which take hold on the foot over the astragalus at one point, and in the plantar arch of the other point of pressure. The foot is placed in the wrench

* Read before the American Orthopedic Association, Washington, D. C., Sept. 20, 1888.

aud the screw tightened, then the arms brought in close proximity until we get a good purchase or grip; then by means of a lever, you can bring an ordinary club-foot, or the kind of club-foot we are familiar with—namely, the relapsed club-foot, the uncured ones—into a good position. I think, sir, the Association will agree with me, that the majority of cases of club-foot we have to treat are those that have been under various forms of treatment, that we fancy we have cured ourselves on former occasions, and find, on looking the matter over again, there is still some defect; we do not get the foot into a good normal position. We get the equinus corrected, perhaps, but the varus is left over, the child walks pigeon-toed, and our attention is not kept closely riveted on the child; we find it rolling, after a few months, on the outer side of the foot, the inner side of the foot coming up. It does not take long to thus acquire one of these relapsing cases.

Dr. Bradford has drawn our attention to this in the presentation of his tarsal plates. Dr. Morton, of Philadelphia, likewise, and now we are getting to look upon the most formidable club-foot as belonging to that class of cases mentioned, to say nothing of what is known as untreated cases of club-foot. None of us, I think, claim to cure club-foot in a short space of time. We all recognize time as an important element in the cure of any case of congenital club-foot. The plan that seems to be most in vogue at the present day, I think, is this, not only among orthopedic surgeons, but general surgeons the world over: to overcome first the varus, either by means of apparatus, which exercise gradual force, or force under ether at one sitting, and then to overcome the equinus by tenotomy, or by manual or mechanical force combined with tenotomy; in other words, our aim is first to make the case one of equino-valgus. I think we all aim to do that. After we have brought about a typical case of equino-valgus, our aim is then to put the foot into a position of calcaneo-valgus. The difficulties we formerly labored under, in producing calcaneus at a single sitting, are not with us at present. I think there is not a single member of this Association, so far as I know, that is afraid to divide the tendo-achillis in a case of congenital club-foot, and get the greatest possible amount of extension or separation of the ends of the tendon. I am now confining my remarks to congenital club-foot. The form known as *spastic* or *hemiplegic* club-foot, some of us still hesitate to make of a case of equinus or a case of calcaneus.

To return to the subject under consideration—namely, the report and description of this wrench for correcting equinus as well as varus, I may say that its principle is practically that of Dr. Bradford's instrument. The object of his apparatus is to grasp the foot in a position that you would naturally take hold with your hands, getting your fingers over the salient points, then twist the foot into shape. I have used the tarsoclast considerably, yet I confess when I saw the apparatus, which I now present to you, a year or so ago, it occurred to me that this was of a much more simple form, one that any one can use more readily. I find considerable difficulty in using Dr. Bradford's instrument, in having students take hold of it, and put in shape; they do not exactly know where to get the points of pressure; the screws and small pads slip, and some of my New York friends have made that objection to it.

The principle of this instrument is so simple that any one can wrench a club-foot into very good shape. I think, however, that the best plan is, before attempting to correct completely, if you have an extreme case of congenital club-foot, to first, by means of manual force, convert an equino-varus into a case of equino-valgus, and leave the foot in position long enough for the bones on the outer side of the foot to become atrophied, and be long enough to hope for some hypertrophy of the inner side of the foot when pressure is removed. Then, after this has been in position long enough, the benefit can be tested in this way: My plan is to leave off plaster dressings for one or two weeks, during which time there is no dressing on the foot. Take the foot and place it on a piece of paper, and make a tracing of the greatest amount of eversion. If, at the end of a week or two, there has been no disposition to return, I begin then the second part of the treatment, viz., divide the tendo-achillis, and bring about both flexion and eversion of the foot by means of the wrench into a case of equino-valgus.

I have used it since last September or October in twenty cases, supplementing it in some instances by manual force.

The following brief abstracts will suffice:

*Case 1*—Male, *æt.*4. Double equino-varus, high degree; never treated. Under ether April 8, 1888, overcame by manual force the varus, and applied plaster of Paris. The dressings were renewed about once a month until August 11th, when the case presented a typical equino-valgus. Tenotomy of tendo-achillis then done without ether and equinus overcome at once. Dressings renewed once or twice, and on each occasion attempt was made by manual force to over-correct the deformity. On September 8th, it was found that the cure was not complete, and under ether the Thomas wrench was employed, completely reducing all deformity. Discharged September 14th, cured; but provided with apparatus which is to be worn for at least one year.

*Case 2*—Male, *æt.* 14 Double talipes equino-varus, from infantile paralysis. Deformity very pronounced, and mid-tarsal region unyielding to manual force. Under ether May 23, 1888, tendo-achillis and tense bands of plantar fascia divided subcutaneously. Then, with Thomas wrench, the arch of foot broken down and a good position obtained; plaster of Paris dressing. June 20th, above procedures repeated with the exception of the tenotomies. Position mainly perfect; same dressing as before. August 29th, operation of left foot, as the right is correct. September 15th, discharged cured, wearing ordinary shoes with soles built higher on outer side.*

*Case 3*—Female, *æt.* 16, with both feet in moderate equino-varus of paralytic origin. Patient very stout, and hence slight amount of deformity made walking quite difficult. Had had several achillotomies, and had worn braces for many years.

May 25, 1888, under ether, tendo-achillis and plantar fascia divided, but deformity could not be overcome until the wrench was employed. With this instrument, deformity over-corrected, and plaster of Paris employed. June 21st, dressing removed and feet in excellent position. A pair of common shoes worn

*November 10th. Recently seen and feet still in excellent position.

with outer side raised a little. At present writing, patient walks easily and expresses herself as fully relieved.

*Case 4*—Female, *æt.9*. Talipes equino-varus from paralysis. Never any treatment until July 27, 1888, when under ether the tendo-achillis was divided and the deformity was over-corrected by the Thomas wrench. August 10th, cure seems complete, yet precaution is taken to build the shoe up and to wear a brace at night.

*Case 5*—Male, *æt.* 11. Double talipes equino-varus (congenital) of high degree. Apparently much bony enlargement of bones of front and outer side of foot; patient walked on outer side of foot. June 6th, under ether, the wrench was employed after division of tendo-achillis, and the deformity was only partially corrected. June 20th, operation repeated. June 30th, a slough was found on outer side of each foot. Plaster was not continued until wound should heal. September 15th, wounds have healed, but there is no further attempt at correcting by means of wrench, as the case seems better suited for cuneiform osteotomy. This case is practically a failure.

*Case 6*—Male, *æt.* 11, with double talipes equino-varus (congenital), relapsed. Cured once or twice and discharged from hospital, but at date of re-admission, March, 1888, the varus was pretty well marked in both feet. Treatment, as in above cases, was resorted to, and a good position obtained. On April 4th, operation repeated. August 17th, patient discharged, with relief say of about 75 per centum, with shoes built up on outer side; the case is to be kept under observation.

*Case 7*—Male, *æt.* 9. Double equino-varus (congenital), relapsing. Treated for many years at hospital, both in and out-door departments. February 17, subjected to treatment as above, and on April 4th operated upon the second, and May 16th the third time. August 14th, discharged, walking with feet squarely on floor and without apparatus. Same precaution, as in above cases, taken to avoid relapse.

*Case 8*—Male, *æt.* 4. Double talipes equino-varus (congenital). Deformity quite marked. Tendo-achillis had been divided five times, and deformity had relapsed after each. January 7th, treated as above, a fair position being obtained. April 4th, operation repeated on both feet. Tenotomy not employed in either instance. June 11th, common pair shoes built up as usual. August 7th, discharged with ability to flex feet to ninety degrees and evert to normal limit. Provided with pair of braces to be worn at night, and to be kept under observation at out-door department.

*Case 9*—Female, *æt.* 11. Double talipes equino-varus (congenital), relapsing. Patient under treatment in out-door department from infancy, with pretty faithful attendance. Deformity not marked, yet feet by no means straight. January 27, 1888, operative treatment as above. February 21, operation repeated, and on July 14th discharged practically cured, the functions of the feet very good. Walks well without apparatus. Usual preliminary measures adopted.

*Case 10*—Male, *æt.* 8. Double talipes equino-varus, congenital. Deformity very marked, yet not extreme. April 4, 1888, operation as above. Two weeks later pneumonia developed and the patient removed from hospital. Seen during the summer, deformity not having relapsed. To be operated upon in the fall if necessary.

*Case 11*—Male, *æt.* 5. Double talipes equino-varus, congenital. Deformity more marked on left side. Has had several tenotomies. January 13th, operation as above, and fairly good position obtained. March 18th, operation repeated; excellent position obtained. May 26th, treatment discontinued. September 14th, patient discharged with usual precautions.

*Case 12*—Male, *æt.* 7. Double talipes equino-varus, paralysis, not very pronounced. July 2d, 1888, operation as above, except that tenotomy was omitted. September 11th, wrench employed again with excellent result. Final result not obtained.

The above list comprises the majority of the cases operated upon, and I regret that I am unable to report final results.

The patients have suffered very little pain after the operation, with one exception, and this is the case where failure is recorded. In no instance have I employed the wrench without the administration of ether.

